# Engineering triterpene metabolism in the oilseed of *Arabidopsis thaliana*

**DOI:** 10.1111/pbi.12984

**Published:** 2018-07-31

**Authors:** Chase Kempinski, Joe Chappell

**Affiliations:** ^1^ Plant Biology Program University of Kentucky Lexington KY USA; ^2^ Department of Pharmaceutical Sciences University of Kentucky Lexington KY USA

**Keywords:** isoprenoid, triterpene, metabolic engineering, oilseed, seed‐specific

## Abstract

Squalene and botryococcene are linear, hydrocarbon triterpenes that have industrial and medicinal values. While natural sources for these compounds exist, there is a pressing need for robust, renewable production platforms. Oilseeds are an excellent target for heterologous production because of their roles as natural storage repositories and their capacity to produce precursors from photosynthetically‐derived carbon. We generated transgenic *Arabidopsis thaliana* plants using a variety of engineering strategies (subcellular targeting and gene stacking) to assess the potential for oilseeds to produce these two compounds. Constructs used seed‐specific promoters and evaluated expression of a triterpene synthase alone and in conjunction with a farnesyl diphosphate synthase (FPS) plus 1‐deoxyxylulose 5‐phosphate synthase (DXS). Constructs directing biosynthesis to the cytosol to harness isoprenoid precursors from the mevalonic acid (MVA) pathway were compared to those directing biosynthesis to the plastid compartment diverting precursors from the methylerythritol phosphate (MEP) pathway. On average, the highest accumulation for both compounds was achieved by targeting the triterpene synthase, FPS and DXS to the plastid (526.84 μg/g seed for botryococcene and 227.30 μg/g seed for squalene). Interestingly, a higher level accumulation of botryococcene (a non‐native compound) was observed when the biosynthetic enzymes were targeted to the cytosol (>1000 μg/g seed in one line), but not squalene (natively produced in the cytosol). Not only do these results indicate the potential of engineering triterpene accumulation in oilseeds, but they also uncover some the unique regulatory mechanisms controlling triterpene metabolism in different cellular compartments of seeds.

## Introduction

Isoprenoids or terpenoids comprise one of the largest classes of natural products with tens of thousands of unique molecules currently identified and estimates that these represent 1%–10% of the total number of possible species. This large catalog of compounds arises, in part, from regio‐ and stereo‐chemistry diversification imposed during their biosynthesis, as well secondary modifications like hydroxylation, acylation, aroylation and glycosylation. While the biosynthesis of this chemical family has recently been reviewed (Hemmerlin *et al*., 2012; Kempinski *et al*., 2015; Vranová *et al*., 2013), isoprenoid biosynthesis proceeds via the generation of the universal 5‐carbon building blocks, isopentenyl diphosphate (IPP) and dimethylallyl diphosphate (DMAPP), which are then polymerized together to yield families of molecules. The condensation of two isoprenyl units, IPP and DMAPP, yields the ten carbon prenyl chain geranyl diphosphate (GPP) by GPP synthase (GPS). GPP is the precursor to monoterpenes. Condensation of two IPPs with one DMAPP generates the fifteen carbon prenyl chain farnesyl diphosphate (FPP) by FPS, and FPP is the precursor to the sesquiterpene family of compounds. The head‐to‐head condensation of two FPPs by squalene synthase gives rise to squalene, the precursor to a complex array of triterpenes including sterols. Three IPP and one DMAPP can be condensed to produce the twenty carbon intermediate geranylgeranyl diphosphate (GGPP) by GGPP synthase (GGPPS), the precursor to the diterpenes. The forty carbon tetraterpenes are derived from the condensation of two GGPPs to yield phytoene by the squalene synthase‐like reaction catalysed by phytoene synthase. In plants, an interesting subcellular division of isoprenoid biosynthesis exists: the MVA pathway resides in the cytosol, whereas the MEP pathway is localized to the plastids, both of which give rise to the initial prenyl units, IPP and DMAPP. However, sesqui‐ and tri‐terpenes are derived from FPP biosynthesized in the cytosol nearly exclusively from the MVA pathway, whereas mono‐, di‐ and tetra‐terpenes are biosynthesized in the plastids from precursors provided by the MEP pathway. These two pathways are understood to normally operate independently of one another, but there have been documented cases of cross‐talk and possible sharing of intermediates or basic prenyl units (Arigoni *et al*., 1997; Flügge and Gao, 2005; Hemmerlin *et al*., 2003; Opitz *et al*., 2014).

Triterpenes arise from the head‐to‐head (1′–1 bond) condensation of two molecules of FPP, and in all eukaryotes this is catalysed by the enzyme squalene synthase (SQS) to produce the symmetrical, linear compound squalene. Squalene can be then be epoxidized by squalene epoxidase (SQE) to produce 2,3‐oxidosqualene, the next committed precursor to all sterols and triterpenes derived from oxidosqualene synthases (OSCs) (Nes, 2011; Xue *et al*., 2012). In the green algae, *Botryococcus braunii* (race B), FPP can be dimerized in an alternative fashion (1′–3 bond) by squalene synthase‐like (SSL) enzymes, to produce the C30 compound, botryococcene. More specifically SSL‐1 generates the presqualene diphosphate intermediate that is then reductively rearranged by SSL‐3 using NADPH to yield botryococcene (Bell *et al*., 2014; Niehaus *et al*., 2011). In *B. braunii*, botryococcene and squalene can be further methylated in a SAM‐dependent manner to generate up to tetramethylated, linear triterpenes (Metzger and Largeau, 2005; Niehaus *et al*., 2012). Both squalene and botryococcene (and especially their carbon‐dense methylated derivatives) are of particular interest because of their capacity to be catalytically or hydro‐cracked and distilled into hydrocarbons that can be used as fuels in current internal combustion engines or as feedstocks for the petrochemical industry (Hillen *et al*., 1982; Tracy *et al*., 2011). In addition, squalene is also of considerable interest due to its use in cosmetics, vaccine adjuvants and purported nutritional benefits (Spanova and Daum, 2011).

Oilseeds comprise an important part of human diets throughout the world and are an important part of the world economy. World oilseed production has increased more than 80 million metric tons from 2011 to 2016 and world demand for oilseed crops for food and industrial uses are expected to increase following increases in population and gross domestic product (USDA, 2016). The increased demand for oilseeds will increase the need to obtain higher yielding or added value seeds in the future, irrespective of whether these seeds will be used for food or industrial uses. *Arabidopsis thaliana* is a member of the Brassicaceae family and is closely related to other important oilseed crops, such as the well‐established and important crop, *Brassica napus* and the emerging oilseed crop, *Camelina sativa* (Bansal and Durrett, 2016; Dalal *et al*., 2015; Li *et al*., 2006). *Arabidopsis* is an excellent model to evaluate seed‐specific engineering schemes due to its physically desirable qualities as a model organism (*i.e*. small stature, rapid lifespan and prolific seed set) and ease of genetic transformation (Bechtold and Pelletier, 1998; Clough and Bent, 1998; Harrison *et al*., 2006; Zhang *et al*., 2006).

Because of their dietary and nutritional roles, attempts at seed‐specific engineering have primarily focused on two main areas: enhancing oil yield/altering oil composition and enhancing/introducing carotenoid biosynthesis in seed tissue (Burkhardt *et al*., 1997; Fujisawa *et al*., 2009; Paine *et al*., 2005; Shewmaker *et al*., 1999; Ye *et al*., 2000). Phytosterol levels have also been enhanced through seed engineering, but this has been demonstrated in *Nicotiana tabacum* rather than an oilseed (Holmberg *et al*., 2002, 2003). The efforts to alter phytosterols in the *N. tabacum* seeds was undertaken by introducing the sterol methyltransferase 1 (SMT1) and/or the 3‐hydroxy‐3‐methylglutaryl‐CoA reductase (HMGR). HMGR is an important rate‐limiting step of the MVA pathway operating in the cytosol. SMT1 has been demonstrated to control phytosterol composition in *N. tabacum* leaves, but not overall phytosterol levels (Sitbon and Jonsson, 2001). In contrast, when over‐expressed in a seed‐specific manner total phytosterols were increased up to 44% in *N. tabacum* (Holmberg *et al*., 2002). Tocopherols and tocotrienols are important nutritional antioxidants, both derived from the MEP pathway, whose levels have also been enhanced through seed‐specific engineering of a homogentisate phytyl transferase (the rate limiting step in tocopherol production; Savidge *et al*., 2002) and over‐expression of a heterologous homogentisic acid geranylgeranyl transferase (the committed step in tocotrienol biosynthesis) in *Zea mays* seeds resulting in up to sixfold elevated levels of tocotrienols and tocopherols (Cahoon *et al*., 2003).

While phytosterols, tocopherols/tocotrienols and carotenoids are all biosynthesized utilizing isoprenoid precursors, up to this point there have been few published attempts to introduce the biosynthesis of ‘non‐native’ isoprenoids in seeds. However, Augustin *et al*. (2015) introduced production of the novel monoterpene, (4*S*)‐limonene and the novel sesquiterpene, (+)‐δ‐cadinene, into *Camelina* seeds by expressing limonene synthase (LS) and (+)‐δ‐cadinene synthase (CDNS). Each of these genes was under a seed‐specific promoter, *NAPIN* (Josefsson *et al*., 1987) and *GLYCININ* (Nielsen *et al*., 1989) respectively. They did this by targeting the terpene synthase enzymes to the cytosol or the plastid compartments in conjunction with the corresponding prenyltransferase, LS + GPS or CDNS + FPS resulting in 1.5–3 mg of limonene/g seed and 2.4–3.0 mg of cadinene/g seed. They also generated plastid‐targeted constructs that included a DXS in conjunction with the prenyl transferase and terpene synthase which boosted terpene production by approximately twofold to ~5 mg/g seed for both terpenes. Borghi and Xie (2016) also used seed‐specific promoters (*BANYULS* and *FRUITFULL*) to drive LS expression in *Camelina* seeds. They expressed only LS and obtained average titres much lower than that reported by Augustin *et al*. (2015): ~20 ng/g seed for LS under the *BANYULS* promoter. This could partly be explained by the difference in expression patterns of the seed‐specific promoters: *BANYULS* is expressed in the seed coat (Nesi *et al*., 2009), whereas *NAPIN* and *GLYCININ* are expressed in developing embryos (Fernandez *et al*., 1991; Nielsen *et al*., 1989) where each cell type may have critical differences in the availability of metabolic precursors.

We wished to evaluate the feasibility of utilizing an oilseed platform to produce the triterpenes botryococcene and squalene. Because these compounds are pure hydrocarbons it seems that an oil‐rich cell would be a natural repository and it may also have benefits in their extraction methodology for the future—due to the nature of in which oil can be expressed from oilseeds. We built on engineering strategies developed previously (Augustin *et al*., 2015; Jiang *et al*., 2016; Wu *et al*., 2006, 2012) in which we targeted the triterpene synthase, SQS, for squalene biosynthesis or a fusion enzyme of SSL‐1 + SSL‐3 (Niehaus *et al*., 2011) (which will be referred to here as botryococcene synthase [BS]), for botryococcene biosynthesis, to either the cytosol or the plastid to take advantage of IPP/DMAPP derived from the MVA or MEP pathways, respectively. Hence, the work reported here evaluates design principles for engineering efficient, high‐level production of triterpenes in seeds, paving the way for seeds as possible production platforms for high‐value triterpenes.

## Results

### Plastid‐targeted engineering of botryococcene and squalene results in accumulation in seeds, which can be increased by including a heterologous DXS

**Figure 1:**
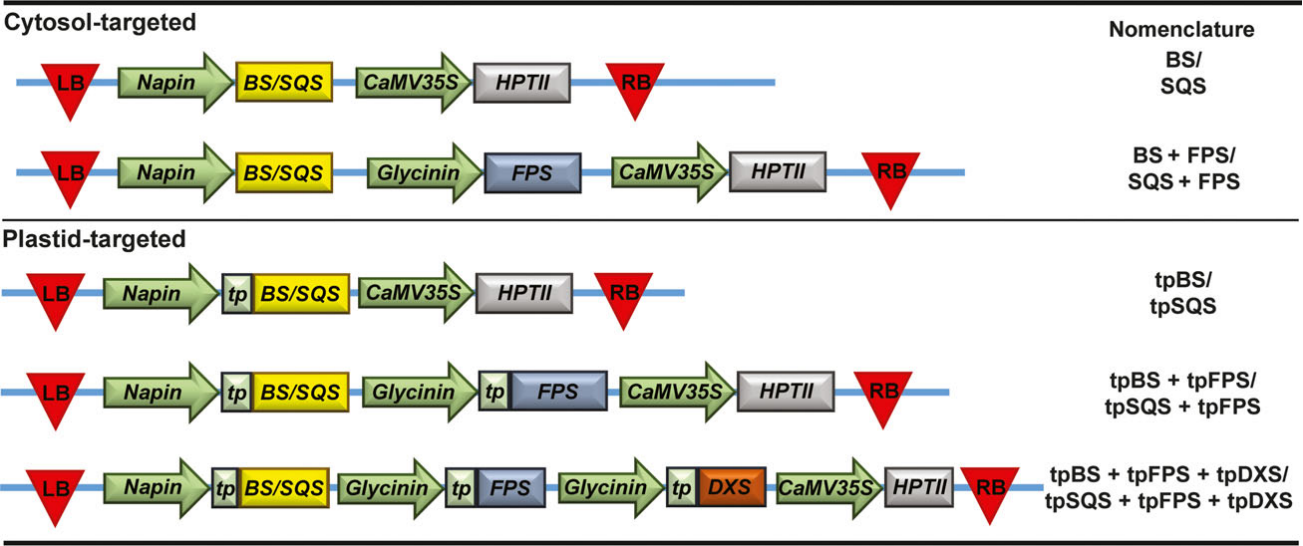
Vector design and nomenclature for seed‐specific engineering in *Arabidopsis*. Vector backbone was that of the pCXUN vector (Chen *et al*., 2009) but modified to remove the monocot‐specific promoter and insert the gene cassettes illustrated. Red triangles represent T‐DNA borders, green arrows represent promoters, rectangles represent coding regions, and the connecting blue line represents the vector backbone. Vectors encoded one triterpene synthase, either BS or SQS. LB, T‐DNA left border; BS, botryococcene synthase; SQS, squalene synthase; HPTII, hygromycin phosphotransferase; CaMV35S, cauliflower mosaic virus 35S promoter; RB, right border; FPS, farnesyl diphosphate synthase; tp, *Arabidopsis* RUBSICO small subunit plastid transit peptide; DXS, 1‐deoxy‐D‐xylulose 5 phosphate synthase.

**Figure 2:**
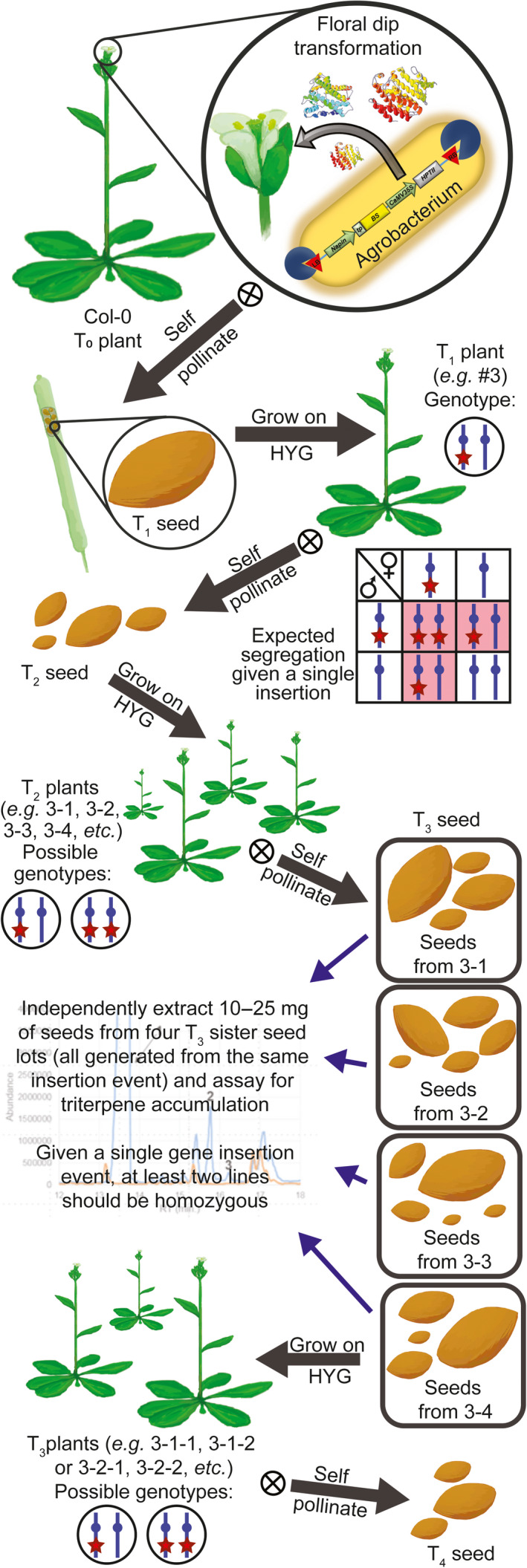
Schematic showing study set up and plant generation designations. All seed generations after transformation were germinated and selected on hygromycin B. The initial plant number is the independent transformation event for that particular construct, with each daughter plant in the following generation indicated by iterative addition of a hyphen and number. Thus, for example plant 3‐2‐5 would be plant 5 (T_3_ generation), a daughter from plant 2 (T_2_ generation), which was a daughter of a T_1_ plant from independent transformation event 3.

**Figure 3:**
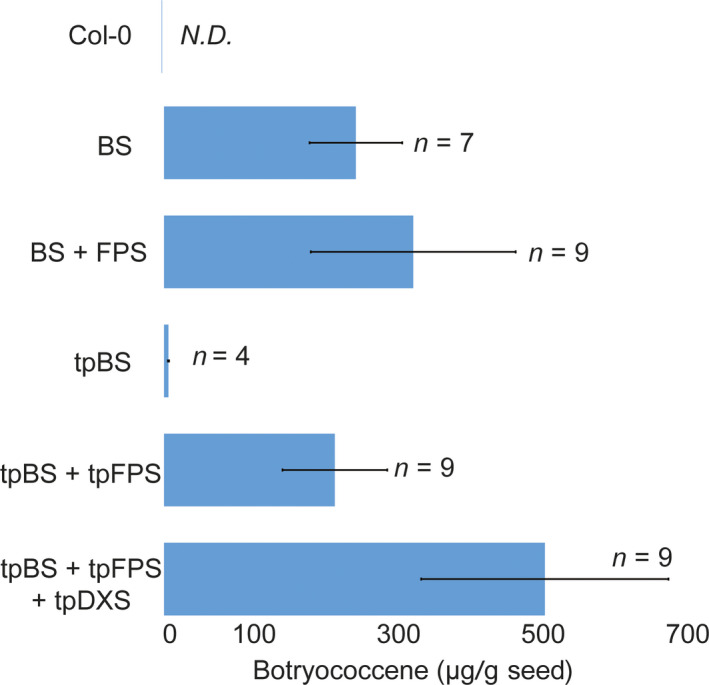
Average botryococcene accumulation (μg/g seed; ±SE) obtained with each construct design. Values are the averages of the highest yielding T_3_ seed lots out of the four assayed (like that shown in Figure S1), representing the average of independently transformed homozygous seed lots obtained with each gene cassette. The number of independently generated transgenic lines (*n*) per construct is noted as well. N.D., none detected.

**Figure 4:**
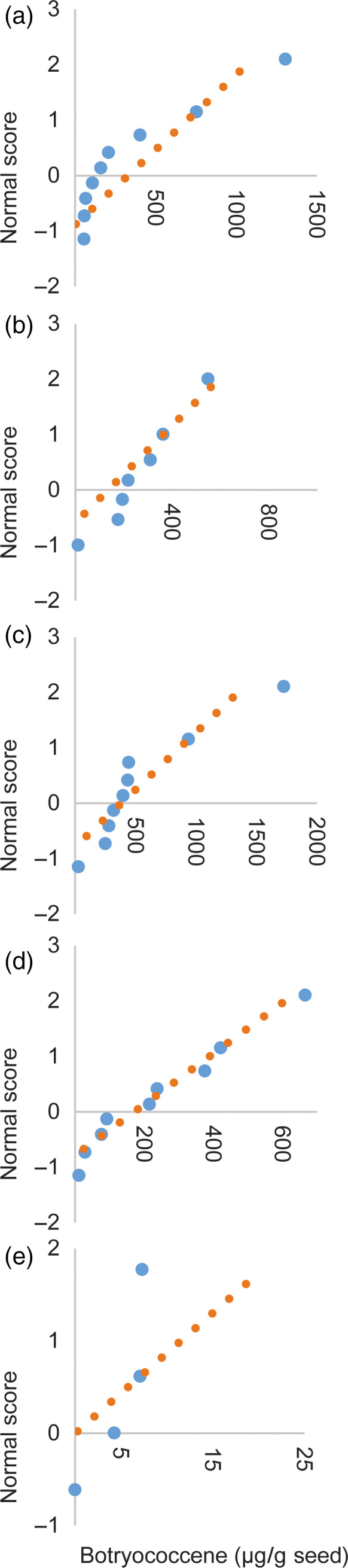
Normal probability plots for the botryococcene accumulation values from independent transformation events for each construct. The accumulation values shown here and used to generate the normal score were the same used to obtain the averages shown in Figure 3. Each construct is shown in its own panel: (a) *BS *+ *FPS*, (b) *BS*, (c) *tpBS *+ *tpFPS *+ *tpDXS*, (d) *tpBS *+ *tpFPS*, (e) *tpBS*. If the values are representative of a normal distribution they should fall along the orange dotted line. By assessing if the triterpene accumulation follows a normal distribution we can presume that we have assayed enough independent transgenic events to determine if the average values of accumulation are representative of the true average amount of product that is obtainable with a particular engineering construct.

**Figure 5:**
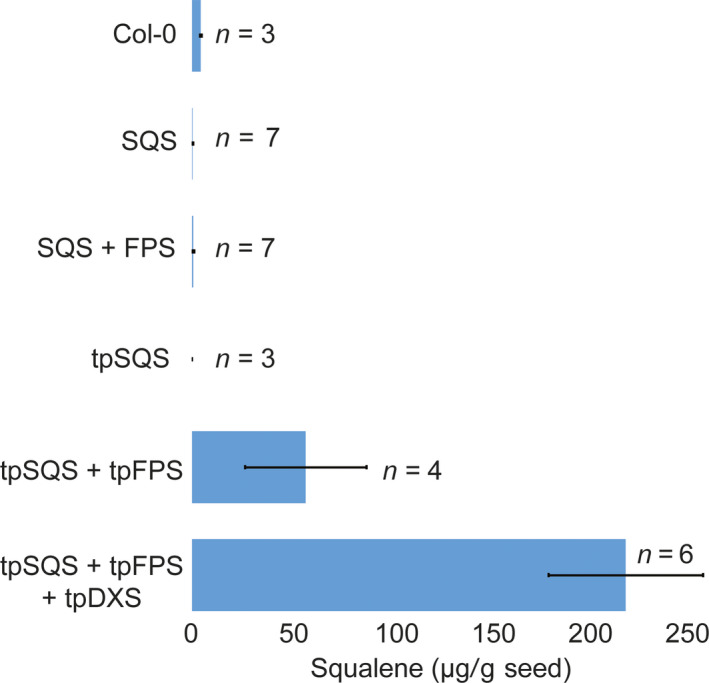
Average squalene accumulation (μg/g seed; ±SE) obtained with each construct design. Values are the averages of the highest yielding T_3_ seed lots out of the four assayed for each independent event shown in Figure S2. They should represent the average of independently transformed homozygous seed lots obtained with each gene cassette. The number of independently generated transgenic lines (*n*) examined is noted, as for three control samples for Col‐0.

We designed constructs that introduced a heterologous triterpene synthase only (BS or SQS for the cytosol and tpBS or tpSQS for the plastid) or in combination with a heterologous FPS (BS + FPS, tpBS + tpFPS, SQS + FPS, tpSQS + tpFPS), as well as adding a plastid‐targeted heterologous DXS into the plastid‐targeted constructs (tpBS + tpFPS + tpDXS or tpSQS + tpFPS + tpDXS) (see Figure 1 for construct schematics). These enzymes were all under their own seed‐specific promoter and transformed into *Arabidopsis* (Figure 2). We allowed plants to self and examined segregation ratios of T_2_ plants germinated on hygromycin B (Tables S1 and S2), and moved resistant plants to soil. The T_3_ seed lots that these individual T_2_ plants produced were screened for triterpene accumulation (see Figure 2 for a diagram of the experimental design). We observed botryococcene accumulation in plants with biosynthesis targeted to the cytosol and plastid, with the highest amounts on average being in the plastid in conjunction with FPS and DXS (Figure 3). To help verify that we assayed enough independently generated lines to account for genomic position effect variation in transgene expression, we created normal score plots for botryococcene accumulating lines (Figure 4). We hypothesized that if botryococcene accumulation truly represented the capability to redirect isoprenoid metabolism in these conditions, then we would expect normal distributions in the botryococcene titers of the populations examined. This would account for lower and higher than expected expression amounts due to position effect variation (or even multiple gene copies). In contrast to botryococcene, we only observed squalene accumulation when SQS was targeted to the plastid in conjunction with FPS, but similar to botryococcene, the highest average titres were observed in the tpSQS + tpFPS + tpDXS engineered lines (Figure 5).

Engineering the triterpene synthases with a heterologous FPS into the plastid resulted in substantial accumulation for both botryococcene and squalene (Figures 3 and 5); although squalene yields were lower than that for botryococcene. Accumulation of both triterpenes in the plastid was dependent on the inclusion of a co‐expressed FPS which is consistent with previous reports (Augustin *et al*., 2015; Jiang *et al*., 2016; Wu *et al*., 2006, 2012). This is due to very low levels of endogenous FPP being produced within the plastid. While there was some botryococcene accumulation (average 6.22 ± 0.851 μg/g seed) in seeds engineered with only tpBS (where none was expected) this could be due to low levels of FPP in the plastid or mis‐targeted enzyme (which could access endogenous FPP produced elsewhere within the cell) which is similar to the results observed by Jiang *et al*. (2016) where they expressed tpBS in *N. tabacum* leaves. In order to verify the seed‐specific nature of our constructs, we extracted several T_1_ plant lines’ leaf tissue and assayed for botryococcene accumulation (we chose botryococcene as it is a foreign molecule whereas squalene would be native to the leaf tissue and a small fraction should be present). In some of the T_1_ lines assayed we did see botryococcene accumulating in the leaves, but only in very minor amounts (Table 1). We also observed minor ectopic expression of the various transgenes in leaf tissue using RT‐PCR but none of this expression followed a specific pattern and appeared stochastic, which we interpret as probably due to position‐dependent effects (data not shown).

**Table 1:** Botryococcene accumulation in *Arabidopsis* leaf tissue of T_1_ lines that were transformed with the indicated genetic construct. The number of independent lines extracted is indicated and the number of those independent lines which accumulated botryococcene is shown. The average (μg/g FW) of those lines which accumulated botryococcene is also presented

Engineered enzymes	T_1_ lines extracted	T_1_ lines with botryococcene	Average (μg/g FW)	SD	SE
BS	18	8	1.37	0.94	0.33
BS + FPS	18	1	3.03	NA	NA
tpBS + tpFPS + tpDXS	9	0	ND	NA	NA

It has been previously reported that DXS may be a rate‐limiting step in carbon flux through the MEP pathway (Hemmerlin *et al*., 2012; Paetzold *et al*., 2010; Wright *et al*., 2014) and isoprenoid engineering in the plastids of the related oilseed plant, *Camelina sativa*, showed increased levels of target isoprenoids with the addition of a co‐expressed DXS (Augustin *et al*., 2015). Because of these observations, we also introduced a plastid‐targeted DXS (*tpDXS*) into the plastid‐targeted gene constructs of *tpBS* + *tpFPS* and *tpSQS* + *tpFPS*. Similar to what was previously seen with seed engineering in *Camelina*, including tpDXS caused an increase in target triterpenoid accumulation for both squalene and botryococcene. The inclusion of tpDXS seemed to boost triterpene content slightly over twofold for both types of triterpene, again with botryococcene accumulating to a higher amount than squalene (~500 μg botryococcene/g seed vs. ~200 μg squalene/g seed; Figures S1 and S2).

### Cytosolic‐targeted engineering in seeds results in botryococcene accumulation, but only marginal squalene accumulation

In order to further evaluate the capabilities of producing linear triterpene in oilseeds we also directed our enzymes into the cytosol to utilize endogenous, MVA pathway produced, FPP. We also introduced genetic cassettes that co‐expressed an FPS enzyme which could utilize IPP/DMAPP produced by the MVA pathway to increase the total FPP available to the triterpene synthase. We observed substantial accumulation of botryococcene in the cytosol with BS alone (~250 μg/g seed) and this increased on average with inclusion of an FPS (~325 μg botryococcene/g seed; Figure 3). Although the inclusion of FPS did not appear to universally increase the amount of botryococcene in the cytosol in all the transgenic lines evaluated, the maximum capacity for botryococcene biosynthesis using MVA‐derived carbon was improved (Figure S1, compare A and B). These results were unexpected given previous work on engineering botryococcene in the cytosol of *N. tabacum* leaves (Jiang *et al*., 2016). However, engineering of the FPP‐derived sesquiterpene, (+)‐δ‐cadinene, in the *Camelina* seeds did result in substantial product accumulation (400–500 μg/g seed), although this was 10‐fold lower than their plastidic‐targeting strategy which included co‐expressing the sesquiterpene synthase, FPS and DXS enzymes (Augustin *et al*., 2015).

**Figure 6:**
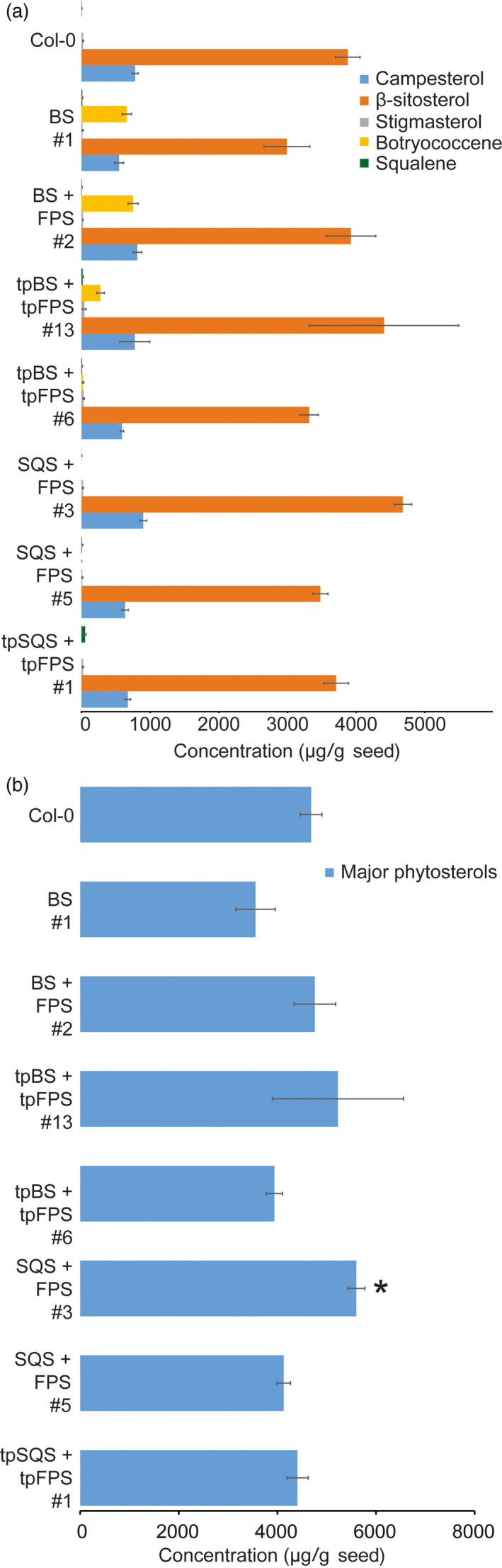
Major phytosterol and triterpene accumulations in T_4_ transgenic lines of the indicated constructs. Values represent the average sterol levels in seed lots from three independent sister T_4_ plants from the same independent transformation event (and six determinations of wild type [Col‐0] seeds). The individual major phytosterols (stigmasterol, β‐sitosterol and campesterol) shown in a) were summed to give the values in (b). The triterpene contents (squalene and botryococcene) of the same transgenic lines are also represented in (a). (±SE,* n *=* *3, *= *P*‐value < 0.05, Student's *t‐*test vs. wild type only conducted for the summed values shown in b)).

Interestingly, attempting to target squalene accumulation to the cytosol did not result in any substantial squalene accumulation beyond what is normally found in wild‐type Col‐0 seeds (~5 μg/g seed; Figure 6). Squalene is normally formed within the cytosol and in association with the Endoplasmic Reticulum (ER) as it is the precursor to phytosterols (and other triterpenes) and thus any squalene formed may be acted upon by squalene monooxygenase and other integral‐membrane, enzymes resulting in accumulation of downstream phytosterols and triterpenes. In order to test this, we examined the levels of major phytosterols (campesterol, stigmasterol and β‐sitosterol) in seeds of T_4_ transgenic lines engineered with BS, BS + FPS, tpBS + tpFPS, SQS + FPS, tpSQS + tpFPS and wild type (Figure 6). Consistent with Nguyen *et al*. (2013), high level expression of a heterologous SQS in *Arabidopsis* seeds resulted in modestly elevated levels of total major phytosterols in only one line examined (Figure 6b). The cytosolic BS and BS + FPS lines accumulating high levels of botryococcene did not show any significant difference in total phytosterols levels compared to wild type. However, there was a slight overall reduction in phytosterol levels in the BS only line examined, suggesting that FPP normally utilized for phytosterol biosynthesis might be rerouted to botryococcene biosynthesis, reducing phytosterol levels in this line. However, the phytosterol levels in the BS + FPS lines accumulating high amounts of botryococcene were approximately the same as wild type, indicating that flux through the MVA pathway was possibly increased in proportion to the IPP/DMAPP diverted to botryococcene biosynthesis. This hints at the metabolic plasticity of the MVA pathway in *Arabidopsis* seeds, which may hinge more on IPP/DMAPP availability than FPP, and can compensate for a drain on IPP/DMAPP by the introduced FPS.

Both of these results suggest a flexibility within the MVA pathway carbon flux to FPP which can be can be utilized by downstream enzymes. However, the capability of the plant to accumulate large amounts of squalene in the cytosol does not seem possible as it is most likely metabolized further, whereas botryococcene can be accumulated as it is not a native substrate for any enzymes present within *Arabidopsis* (or any organism besides *Botryococcus braunii*).

### Accumulation of triterpene has no effect on total seed weight *per se*, but redirection of plastid metabolism might

**Figure 7:**
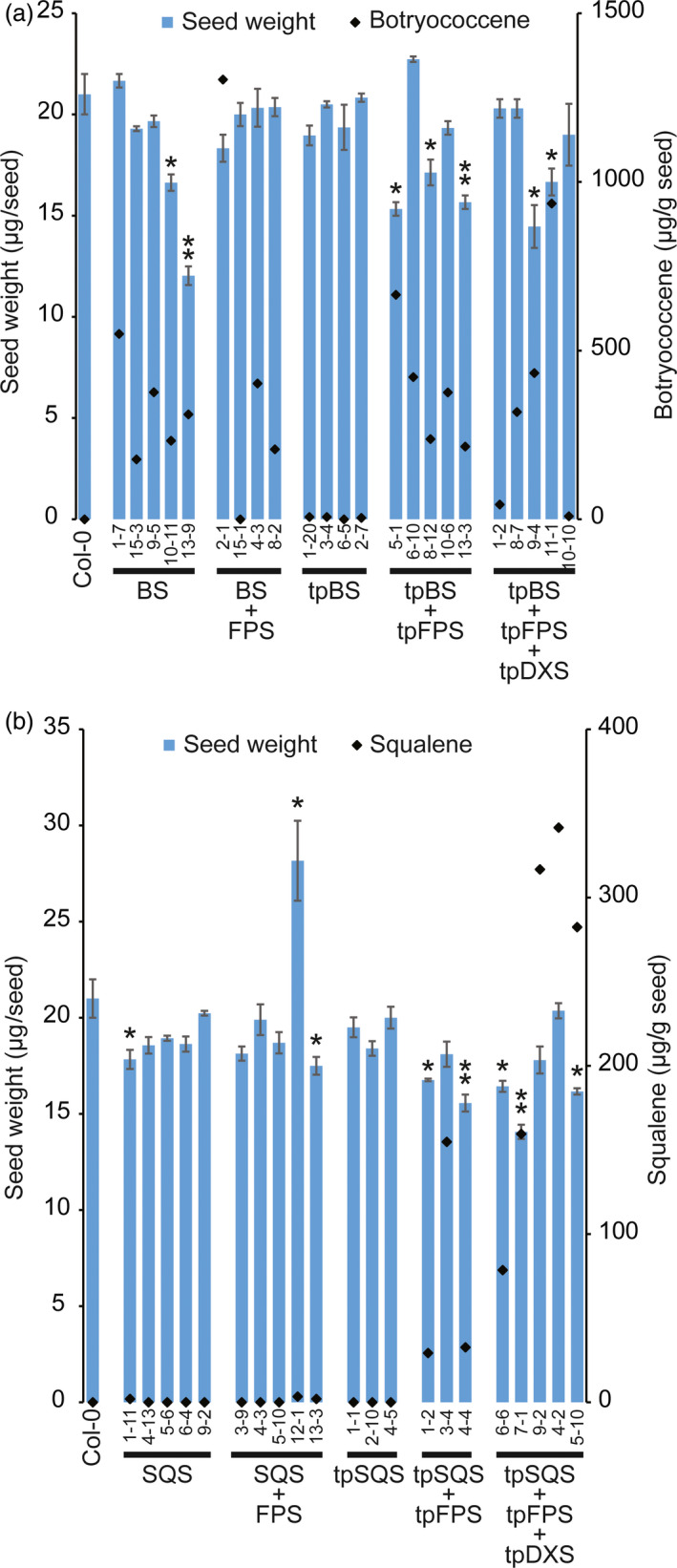
Average seed weights (μg/seed; blue bars) from indicated T_3_ seed lots. Lines engineered for botryococcene are shown in (a) and those engineered for squalene are shown in (b). Values were determined from 100 seeds, *n *=* *3, ±SE, * denotes significance at *P*‐value < 0.05 and ** denotes significance at *P*‐value < 0.01 versus Col‐0, Student's *t*‐test. Black diamonds represent botryococcene (a) and squalene (b) content from a single determination.

Considering the potential applied uses of this engineering, and since seed weight is a critical agronomic yield parameter in such applications, we evaluated if engineering triterpene metabolism and accumulation impacted seed weights in various transgenic T_3_ seed lines. Seed weights from multiple independent transgenic events were determined. It is important to note that seed weight can vary depending on the environmental conditions in which the plants are grown, with light intensity playing an important role in seed oil content (and thus, weight) in *Arabidopsis* (Li *et al*., 2006). The plants in this study were hence grown to minimize differences in light irradiation, which varied less than 10% across a shelf and between the various shelves. Plant positions on shelves were also occasionally rotated. While we attempted to survey homozygous lines to present the most accurate values for our analysis, ambiguous resistance to the hygromycin selection marker occasionally complicated zygosity determinations. This was evident in seed lots from an individual plant that produced multiple phenotypes (Figure S3, bottom). T_3_ seed lines randomly selected for high level triterpene accumulation (Figures S1 and S2) were weighed (except for SQS, SQS + FPS and tpSQS lines which did not show accumulation). As seen in Figure 7, it does not appear that accumulation of either triterpene directly correlates with any impact on seed weight. However, in both engineering strategies a significant reduction in average seed weight was more often observed when engineering was directed to the plastid, and this reduction depended on presence of a plastid‐targeted FPS. However, our analysis of crude seed oil as a percent of seed weight did not show any significant differences when compared to wild type (Figure S4). Some cytosolic‐targeted lines did demonstrate a reduction in seed weight [BS 10‐11 and 13‐9 (Figure 7a) and SQS + FPS 13‐3 and SQS 1‐11 (Figure 7b)]. To check if seed oil content might be altered leading to this altered seed weight we evaluated crude seed oil from select lines (Figure S4). We did not see any significant difference in crude seed oil when expressed as a percent of seed weight in any of the lines, regardless if they exhibited significantly reduced total seed weight (compare selected lines from Figure 7 in Figure S4).

## Discussion

In these experiments we have demonstrated the feasibility of producing the triterpene hydrocarbons, botryococcene and squalene, in the oilseeds of *Arabidopsis*. We obtained the highest average yields when targeting our metabolism to the plastid in conjunction with expression of a putative rate‐limiting step in the MEP pathway, catalysed by DXS. We have also shown the ability to produce botryococcene using a cytosolic‐targeted strategy, but not squalene. We hypothesize that the inability to accumulate squalene in the cytosol is due to its metabolism, either by the conventional sterol biosynthetic machinery or alternative machinery. The latter result could come about by the form of the heterologous expressed squalene synthase used. The SQS used in the current work originates from *Saccharomyces cerevisiae* and its native, carboxy‐terminal membrane‐spanning domain was deleted and hence, encodes for a soluble SQS and not one in association with the ER membrane. The molecular fate of the squalene produced by this soluble SQS enzyme could be entirely different from that generated by ER membrane tethered enzyme. Previous reports indicate the inability of a heterologously expressed SQS to complement and participate in sterol biosynthesis in a cross‐kingdom manner, which may be occurring here (Linscott *et al*., 2016). Nguyen *et al*. (2013) previously reported significantly elevated phytosterols in *Arabidopsis* seeds due to seed‐specific expression of a full‐length, ER‐targeted soybean SQS. In contrast, we observed only a single transgenic event where total sterol content was significantly enhanced with a truncated, soluble SQS. More typically, we did not observe a significant change in total sterol levels in the soluble SQS engineered lines. As stated above, this could be, in part, due to the inability of the introduced *S. cerevisiae* SQS to integrate with the native *Arabidopsis* sterol machinery, which may require protein‐protein interactions that are dictated by plant‐specific motifs present on those proteins (Linscott *et al*., 2016).

A second limitation of our work is that the absolute levels of squalene produced in the cytoplasm might not be sufficient to significantly alter the observable sterol levels. When targeted to the plastid compartment, upwards of 200 μg of squalene per g seed accumulated. If a similar amount of production occurred in the cytosolic engineered lines, this would represent an extra 200 μg in addition to the 3000 μg of total phytosterols. Reliably observing less than a 10% increase in total phytosterols represents a challenge beyond our current technical capabilities. Hence, we conclude that extra squalene produced in transgenic *Arabidopsis* seeds harboring a cytosolic, soluble SQS could be funneled into the phytosterol pools and escape detection because shear mass is not significant, or it could be subject to alternative metabolism (catabolism?) due to its unusual intracellular production.

Equally intriguing, we saw some botryococcene accumulation in lines engineered with only tpBS (no co‐expressed tpFPS), but no squalene accumulation in lines engineered with only tpSQS. Because FPP is not natively produced within the plastid (and believed to be nearly absent in that compartment), these results suggest that botryococcene present in these plants might be the result of a mis‐targeted enzyme (*i.e*. tpBS residing within the cytosol or mitochondria—both locations where FPP is normally produced), similar to results reported by Jiang *et al*. (2016). Other studies have shown the availability of MEP‐derived prenyl units to cytosolic enzymes but not *vice‐versa* (Dudareva *et al*., 2005; Gutensohn *et al*., 2013), hence we do think a plastid targeted SQS could be receiving its substrate (FPP) from an MVA origin. We also cannot eliminate the possibility that a minor amount of FPP could be released from the plastidic GGPS during GGPP formation. However, this possibility also seems unlikely because no squalene was observed in the tpSQS only engineered lines. Hence, the most likely explanation is that a small portion of the tpBS synthesized in the cytoplasm does not find its way to the plastid compartment and thus has cytosolic FPP available for catalysis.

We have demonstrated the ability to produce the linear triterpenes, botryococcene and squalene in the oilseeds of *Arabidopsis thaliana* using various subcellular targeting strategies and gene stacking. Targeting biosynthesis to the plastid in combination with a heterologous DXS gene resulted in the highest average accumulation for both compounds. However, cytosolic botryococcene production was substantial, and illustrates the capacity of the MVA pathway to produce nonnative terpenes. It may be worthwhile in the future to express a soluble, heterologous HMGR in combination with FPS and BS to evaluate this production potential. Similarly, higher levels of production in the plastid might be obtained by expressing the last two MEP pathway enzymes 4‐hydroxy‐3‐methylbut‐2‐enyl‐diphosphate synthase and/or 4‐hydroxy‐3‐methylbut‐2‐enyl‐diphosphate reductase, as these have been speculated to also control MEP flux (Banerjee and Sharkey, 2014). A striking difference between our work and that of engineering sesquiterpene metabolism in *Camelina* (Augustin *et al*., 2015) is the higher titres of sesquiterpene (also FPP‐derived) they were able to obtain in both the cytosol and plastid compared to what we have observed with our triterpene engineering in *Arabidopsis*. Because their experiments also involved seed‐specific promoters, inherent physiological differences might account for the production differences between these two species. Considering this, further seed engineering efforts may wish to evaluate a broader array of oilseed species (and accessions) to identify those best suited for each isoprenoid engineering project. Regardless, because of the ease to store and extract seeds, seeds appear to be an ideal tissue for just‐in‐time manufacturing of highly desired, commercially relevant chemical targets.

## Experimental procedures

### Plant growth and transformation

All transgenic lines were generated in the Col‐0 ecotype background. Plants were grown on Pro‐Mix BX soil (PremierTech) in 8 cm wide × 8 cm long × 10 cm deep pots in flats and were sub‐irrigated as necessary with fertilizing approximately every other watering using 20 : 20 : 20 (N : P : K) general purpose fertilizer (The Scotts Company) at a nitrogen amount of 300 ppm. Plants were grown under 16 h light 8 h dark cycles using Sylvania OCTRON 6500K bulbs (Sylvania), with an approximate photosynthetically active range light intensity of 50 μmol/m^2^/s.

Plants (Col‐0) were transformed with *Agrobacterium tumefaciens* strain GV3101 harboring the desired genetic construct using the floral dip method as previously described (Zhang *et al*., 2006). T_1_ seeds were collected and screened on 20 μg/mL hygromycin B (Invivogen) on 1x MS salts (Phytotech; Murashige and Skoog, 1962) without sucrose using the method described by Harrison *et al*. (2006) under the same growth conditions as above. Subsequent generations were screened using this method as well (see Figure 2 and Tables S1 and S2).

### Generation of transformation constructs

All plant transformation vectors were based on the pCXUN vector backbone as described by Chen *et al*. (2009) which contains the hygromycin phosphotransferase II (*HPTII)* gene under the constitutive cauliflower mosaic virus 35S promoter (*CaMV35S*) encoding hygromycin resistance. The C‐terminal truncated *Saccharomyces cerevisiae SQS* (GenBank ID: NM001179321) and the *Gallus gallus FPS* (Tarshis *et al*., 1994; GenBank ID: 425061) genes utilized here were the same utilized in Wu *et al*. (2012) and Jiang *et al*. (2016) for *Nicotiana tabacum* transformation. The plastid‐transit peptide used in these experiments is derived from the *Arabidopsis* RUBISCO small subunit protein (GenBank ID: NM23202; Lee *et al*., 2006). The *BS* construct was a fusion of the *B. braunii SSL‐1* and *SSL‐3* coding sequences as generated by Niehaus *et al*. (2011). The constructs with *DXS* were generated by taking an *Escherichia coli DXS* gene (GenBank ID: NC000913) and having it codon optimized for plant expression (Addgene). The seed‐specific promoters, *NAPIN* from *Brassica napus* (Josefsson *et al*., 1987), and *GLYCININ1* from *Glycine max* (Nielsen *et al*., 1989; Sims and Goldberg, 1989), both kindly provided by Dr. Edgar Cahoon (University of Nebraska). For full construct generation see supplemental experimental procedures.

All vectors were sequence verified using BigDye terminator sequencing reactions (Agilent) and sequenced at the University of Kentucky's Advanced Genetic Technology Center. The vectors were transformed into *A. tumefaciens* strain GV3101 which was used for plant transformation.

### Normal score calculation

The botryococcene accumulation amounts obtained from each construct were evaluated to see if they followed a normal distribution. To do this, the highest botryococcene accumulating T_3_ lines (*n*) from independent transformation events (the same that were used to generate the averages shown in Figure 3) were used to create plotting points from 0 to 1 by listing the *n* accumulation amounts in ascending order and taking the rank in this list of *n* and using it in the formula: (rank + (1/6))/(*n *+* *1/3). The resulting value was used to generate a normal score in Excel (Microsoft) with the NORMSINV function. This normal score was plotted on the *y*‐axis with the corresponding accumulation value used to obtain the normal score plotted on the *x*‐axis (Figure 4). A linear trendline was fitted to the data to show the expected normal distribution of the data.

### Metabolite extraction and quantification

The triterpenes squalene and botryococcene were extracted from leaves by harvesting tissue in a pre‐weighed 4 mL glass vial and grinding in liquid nitrogen. The tissue was allowed to warm to room temperature then weighed to obtain the fresh weight. A 1 : 1 : 1 mixture of hexane:acetone:water (1 mL each) was added to the sample (plus 100 μL of a 100 ng/μL hexadecane internal standard). For seed tissue, the seeds were weighed (~10–25 mg) moved to a clean mortar, 100 μL of a 100 ng/μL hexadecane internal standard was added, followed by 1 mL acetone and 1 mL water. The seeds were then ground using a clean pestle and 1 mL of hexane was then added and the slurry was ground further before being moved to a 4 mL glass vial. Another 1 mL of hexane was added to the mortar to rinse and collect any remaining seed tissue and combined with the above slurry. The remainder of the extraction process was the same for both types of tissue. The solutions was allowed to shake (~220 rpm) at room temperature, in a horizontal position, for at least 20 min. The phases were then allowed to separate (or centrifuged briefly to separate the organic and aqueous phases), and the hexane phase was aspirated off and saved. Two more rounds of extraction with 1 mL hexane were completed and pooled with the initial extract. This n‐hexane extraction was dried under nitrogen gas, resuspended in iso‐octane and transferred to a GC vial. Samples were analysed on either an Agilent 7890 GC (HP‐5MS column, 30 m × 0.25 mm, 0.25 μm film, 250 °C inlet temperature; oven temperature was 150 °C for 1 : 00 min, then 10 °C/min to 280 °C at, then 5 °C/min to 310 °C for 1 : 00 min; 0.9 mL/min He flow rate) connected to a 5975C Agilent mass spectrometer (run in positive ionization mode, 70 eV, scanning 50–500 amu) or an Agilent 7890 GC (HP‐5 column, 30 m × 0.32 mm, 0.25 μm film; 60 °C for 1 : 00 min, then 30 °C/min to 230 °C, then 2 °C/min to 280 °C; 5.7494 mL/min He flow rate) equipped with a flame ionization detector. Botryococcene and squalene in experimental samples was verified by comparing to external standards. Triterpene accumulation was calculated based on an external standard curve run in tandem with experimental samples.

Sterol levels in seeds were analysed by grinding tissue (~100 mg) in a mortar and pestle then moving to a pre‐weighed 24 mL glass vial and weighed. The samples were saponified and the total sterols (and triterpenes) were extracted as described by Du and Ahn (2002). The hexane extraction was dried to completion under nitrogen gas and samples were derivatized using a 1 : 1 mixture of pyridine and MSTFA + 1% TMCS at 50 °C for 1 h. Samples were analysed by GC‐MS (same parameters as above) except the oven temperature was 200 °C initially, 10 °C/min to 270 °C, then 3 °C/min to 320 °C and held at 320 °C for 10 min. Major sterols were compared to known standards that had been derivatized as above.

### Seed weight and crude oil calculations

Determination of seed weights was conducted by averaging the weight of three replicates of 100 seeds from a seed lot. In order to determine the wild‐type (Col‐0) seed weight, three replicates were used from wild‐type seed lots bulked up at different times from various areas of the growth carts. This should reflect the inherent variation in the seed weights due to environmental factors. Seed crude oil was determined by conducting an extraction for triterpene as described above, but the hexane phases were collected in a pre‐weighed vial and after all phases had been collected the hexane was evaporated and the remaining oil in the vial was weighed. This is what we denoted as the seed crude oil.

## Conflicts of interest

Intellectual property has been secured by the University of Kentucky for some of this work and CK and JC are actively pursuing commercialization of this technology.

## Author contributions

J.C. and C.K. conceived the research plan and wrote the article; C.K. conducted the experiments.

## Supporting information


**Table S1** Segregation ratios for T_2_ *Arabidopsis* plants engineered with various squalene biosynthesis constructs.
**Table S2** Segregation ratios for T_2_ *Arabidopsis* plants engineered with various botryococcene biosynthesis constructs.
**Figure S1** Botryococcene content (μg/g seed) of four independent T_3_ seed lots.
**Figure S2** Squalene content (μg/g seed) of four independent T_3_ seed lots.
**Figure S3** Example of phenotypes complicating segregation analyses.
**Figure S4** Crude seed oil (% seed weight) and triterpene values from indicated T_3_ seed lots.
