# Supplementary material for: Engineering triterpene metabolism in the oilseed of Arabidopsis thaliana
Source: Plant Biotechnol J. 2018 Jul 31;17(2):386–96. doi: 10.1111/pbi.12984 (PMC6335079; doi:10.1111/pbi.12984)
Supplement: Supplementary file 1 — Table S1 Segregation ratios for T2 Arabidopsis plants engineered with various squalene biosynthesis constructs. Table S2 Segregation ratios for T2 Arabidopsis plants engineered with various botryococcene biosynthesis constructs. Figure S1 Botryococcene content (μg/g seed) of four independent T3 seed lots. Figure S2 Squalene content (μg/g seed) of four independent T3 seed lots. Figure S3 Example of phenotypes complicating segregation analyses. Figure S4 Crude seed oil (% seed weight) and triterpene values from indicated T3 seed lots. [file PBI-17-386-s001.docx]

Supporting information

Table S1. Segregation ratios for T_2_ *Arabidopsis* plants engineered with various squalene biosynthesis constructs. Plants were grown on hygromycin selection and the corresponding Χ^2^ test to determine if the observed hygromycin resistant and sensitive lines follow a single-gene insertion segregation pattern (3:1).

| ***SQS*** | | (df=1, a=0.05, Χ^2^ value from table: 3.841) | | | |  |  |
| --- | --- | --- | --- | --- | --- | --- | --- |
| Line | Observed Positive | Observed Negative | Total plants examined | Expected positive | Expected Negative | Χ^2^ value | Meets single gene expectation? |
| 1 | 59 | 17 | 76 | 57 | 19 | 0.281 | Yes |
| 4 | 106 | 21 | 127 | 95.25 | 31.75 | 4.853 | No |
| 5 | 90 | 17 | 107 | 80.25 | 26.75 | 4.738 | No |
| 6 | 100 | 21 | 121 | 90.75 | 30.25 | 3.771 | Yes |
| 8 | 77 | 26 | 103 | 77.25 | 25.75 | 0.003 | Yes |
| 9 | 87 | 12 | 99 | 74.25 | 24.75 | 8.758 | No |
| 11 | 87 | 14 | 101 | 75.75 | 25.25 | 6.683 | No |
| 12 | 95 | 20 | 115 | 86.25 | 28.75 | 3.551 | Yes |
| 14 | 80 | 13 | 93 | 69.75 | 23.25 | 6.025 | No |
| 15 | 75 | 14 | 89 | 66.75 | 22.25 | 4.079 | No |
| 17 | 71 | 23 | 94 | 70.5 | 23.5 | 0.014 | Yes |
| ***SQS + FPS*** | | |  |  |  |  |  |
| Line | Observed Positive | Observed Negative | Total plants examined | Expected positive | Expected Negative | Χ^2^ value | Meets single gene expectation? |
| 1 | 75 | 12 | 87 | 65.25 | 21.75 | 5.828 | No |
| 2 | 84 | 16 | 100 | 75 | 25 | 4.320 | No |
| 3 | 65 | 17 | 82 | 61.5 | 20.5 | 0.797 | Yes |
| 4 | 70 | 12 | 82 | 61.5 | 20.5 | 4.699 | No |
| 5 | 83 | 26 | 109 | 81.75 | 27.25 | 0.076 | Yes |
| 6 | 44 | 4 | 48 | 36 | 12 | 7.111 | No |
| 7 | 77 | 16 | 93 | 69.75 | 23.25 | 3.014 | Yes |
| 8 | 64 | 9 | 73 | 54.75 | 18.25 | 6.251 | No |
| 10 | 49 | 10 | 59 | 44.25 | 14.75 | 2.040 | Yes |
| 12 | 45 | 13 | 58 | 43.5 | 14.5 | 0.207 | Yes |
| 13 | 74 | 6 | 80 | 60 | 20 | 13.067 | No |
| 14 | 70 | 17 | 87 | 65.25 | 21.75 | 1.383 | Yes |
| ***tpSQS*** | |  | | | |  |  |
| Line | Observed Positive | Observed Negative | Total plants examined | Expected positive | Expected Negative | Χ^2^ value | Meets single gene expectation? |
| 1 | 63 | 20 | 83 | 62.25 | 20.75 | 0.036 | Yes |
| 2 | 66 | 20 | 86 | 64.5 | 21.5 | 0.140 | Yes |
| 4 | 108 | 23 | 131 | 98.25 | 32.75 | 3.870 | No |
| ***tpSQS + tpFPS*** | | |  |  |  |  |  |
| Line | Observed Positive | Observed Negative | Total plants examined | Expected positive | Expected Negative | Χ^2^ value | Meets single gene expectation? |
| 1 | 41 | 13 | 54 | 40.5 | 13.5 | 0.025 | Yes |
| 2 | 7 | 3 | 10 | 7.5 | 2.5 | 0.133 | Yes |
| 4 | 72 | 17 | 89 | 66.75 | 22.25 | 1.652 | Yes |
| ***tpSQS + tpFPS + tpDXS*** | | | |  |  |  |  |
| Line | Observed Positive | Observed Negative | Total plants examined | Expected positive | Expected Negative | Χ^2^ value | Meets single gene expectation? |
| 2 | 35 | 11 | 46 | 34.5 | 11.5 | 0.029 | Yes |
| 3 | 53 | 13 | 66 | 49.5 | 16.5 | 0.990 | Yes |
| 4 | 61 | 24 | 85 | 63.75 | 21.25 | 0.475 | Yes |
| 5 | 64 | 24 | 88 | 66 | 22 | 0.242 | Yes |
| 6 | 89 | 22 | 111 | 83.25 | 27.75 | 1.589 | Yes |
| 7 | 67 | 12 | 79 | 59.25 | 19.75 | 4.055 | No |
| 8 | 56 | 17 | 73 | 54.75 | 18.25 | 0.114 | Yes |
| 9 | 70 | 15 | 85 | 63.75 | 21.25 | 2.451 | Yes |

Table S2. Segregation ratios for T_2_ *Arabidopsis* plants engineered with various botryococcene biosynthesis constructs. Plants were grown on hygromycin selection and the corresponding Χ^2^ test to determine if the observed hygromycin resistant and sensitive lines follow a single-gene insertion segregation pattern (3:1).

| ***BS*** | | (df=1, a=0.05, Χ^2^ value from table: 3.841) | | | |  |  |
| --- | --- | --- | --- | --- | --- | --- | --- |
| Line | Observed Positive | Observed Negative | Total plants examined | Expected positive | Expected Negative | Χ^2^ value | Meets single gene expectation? |
| 1 | 36 | 14 | 50 | 37.5 | 12.5 | 0.240 | Yes |
| 2 | 26 | 14 | 40 | 30 | 10 | 2.133 | Yes |
| 4 | 44 | 10 | 54 | 40.5 | 13.5 | 1.210 | Yes |
| 5 | 35 | 9 | 44 | 33 | 11 | 0.485 | Yes |
| 6 | 45 | 12 | 57 | 42.75 | 14.25 | 0.474 | Yes |
| 7 | 35 | 11 | 46 | 34.5 | 11.5 | 0.029 | Yes |
| 8 | 15 | 24 | 39 | 29.25 | 9.75 | 27.769 | No |
| 9 | 30 | 7 | 37 | 27.75 | 9.25 | 0.730 | Yes |
| 10 | 41 | 11 | 52 | 39 | 13 | 0.410 | Yes |
| 11 | 53 | 11 | 64 | 48 | 16 | 2.083 | Yes |
| 12 | 29 | 9 | 38 | 28.5 | 9.5 | 0.035 | Yes |
| 13 | 34 | 8 | 42 | 31.5 | 10.5 | 0.794 | Yes |
| 14 | 36 | 9 | 45 | 33.75 | 11.25 | 0.600 | Yes |
| 15 | 35 | 10 | 45 | 33.75 | 11.25 | 0.185 | Yes |
| 16 | 72 | 28 | 100 | 75 | 25 | 0.480 | Yes |
| ***BS + FPS*** | | |  | | | |  |
| Line | Observed Positive | Observed Negative | Total plants examined | Expected positive | Expected Negative | Χ^2^ value | Meets single gene expectation? |
| 2 | 28 | 9 | 37 | 27.75 | 9.25 | 0.009 | Yes |
| 4 | 28 | 10 | 38 | 28.5 | 9.5 | 0.035 | Yes |
| 6 | 38 | 11 | 49 | 36.75 | 12.25 | 0.170 | Yes |
| 7 | 35 | 13 | 48 | 36 | 12 | 0.111 | Yes |
| 8 | 43 | 10 | 53 | 39.75 | 13.25 | 1.063 | Yes |
| 9 | 47 | 10 | 57 | 42.75 | 14.25 | 1.690 | Yes |
| 10 | 41 | 11 | 52 | 39 | 13 | 0.410 | Yes |
| 11 | 54 | 10 | 64 | 48 | 16 | 3.000 | Yes |
| 12 | 32 | 14 | 46 | 34.5 | 11.5 | 0.725 | Yes |
| 13 | 46 | 3 | 90 | 67.5 | 22.5 | 23.748 | No |
| 15 | 51 | 6 | 57 | 42.75 | 14.25 | 6.368 | No |
| 16 | 47 | 10 | 57 | 42.75 | 14.25 | 1.690 | Yes |
| 17 | 52 | 16 | 68 | 51 | 17 | 0.078 | Yes |
| 18 | 49 | 14 | 63 | 47.25 | 15.75 | 0.259 | Yes |
| 19 | 50 | 18 | 68 | 51 | 17 | 0.078 | Yes |
| ***tpBS*** | |  | | | |  |  |
| Line | Observed Positive | Observed Negative | Total plants examined | Expected positive | Expected Negative | Χ^2^ value | Meets single gene expectation? |
| 1 | 61 | 33 | 94 | 70.5 | 23.5 | 5.121 | No |
| 2 | 26 | 14 | 40 | 30 | 10 | 2.133 | Yes |
| 3 | 67 | 14 | 81 | 60.75 | 20.25 | 2.572 | Yes |
| 4 | 33 | 9 | 42 | 31.5 | 10.5 | 0.286 | Yes |
| 5 | 52 | 13 | 65 | 48.75 | 16.25 | 0.867 | Yes |
| 6 | 46 | 17 | 63 | 47.25 | 15.75 | 0.132 | Yes |
| 7 | 63 | 17 | 80 | 60 | 20 | 0.600 | Yes |
| 8 | 58 | 16 | 74 | 55.5 | 18.5 | 0.450 | Yes |
| 9 | 60 | 24 | 84 | 63 | 21 | 0.571 | Yes |
| 10 | 65 | 22 | 87 | 65.25 | 21.75 | 0.004 | Yes |
| ***tpBS + tpFPS*** | | |  |  |  |  |  |
| Line | Observed Positive | Observed Negative | Total plants examined | Expected positive | Expected Negative | Χ^2^ value | Meets single gene expectation? |
| 3 | 105 | 58 | 163 | 122.25 | 40.75 | 9.736 | No |
| 5 | 203 | 61 | 264 | 198 | 66 | 0.505 | Yes |
| 6 | 84 | 36 | 120 | 90 | 30 | 1.600 | Yes |
| 8 | 69 | 27 | 96 | 72 | 24 | 0.500 | Yes |
| 10 | 34 | 7 | 41 | 30.75 | 10.25 | 1.374 | Yes |
| 13 | 71 | 19 | 90 | 67.5 | 22.5 | 0.726 | Yes |
| 14 | 39 | 28 | 67 | 50.25 | 16.75 | 10.075 | No |
| 15 | 75 | 18 | 93 | 69.75 | 23.25 | 1.581 | Yes |
| 17 | 127 | 45 | 172 | 129 | 43 | 0.124 | Yes |
| ***tpBS + tpFPS + tpDXS*** | | | |  |  |  |  |
| Line | Observed Positive | Observed Negative | Total plants examined | Expected positive | Expected Negative | Χ^2^ value | Meets single gene expectation? |
| 1 | 148 | 50 | 198 | 148.5 | 49.5 | 0.007 | Yes |
| 2 | 59 | 23 | 82 | 61.5 | 20.5 | 0.407 | Yes |
| 3 | 41 | 38 | 79 | 59.25 | 19.75 | 22.485 | No |
| 6 | 105 | 53 | 158 | 118.5 | 39.5 | 6.152 | No |
| 7 | 7 | 38 | 45 | 33.75 | 11.25 | 84.807 | No |
| 8 | 43 | 31 | 74 | 55.5 | 18.5 | 11.261 | No |
| 9 | 66 | 25 | 91 | 68.25 | 22.75 | 0.297 | Yes |
| 10 | 29 | 12 | 41 | 30.75 | 10.25 | 0.398 | Yes |
| 11 | 69 | 33 | 102 | 76.5 | 25.5 | 2.941 | Yes |

**Figure S1**. Botryococcene content (μg·g seed^-1^) of four independent T_3_ seed lots. These T_3_ seed lots were derived from four sister T_2_ plants from an independent transformation event of the designated construct (see Figure 2 for schematic for how tissue was generated). Bars represent a single determination from 10-25 mg of seeds. Three independently generated lines are shown for each construct, representing low, medium, and high accumulators. Engineered enzymes are: a) BS, b) BS + FPS, c) tpBS, d) tpBS + tpFPS, e) tpBS + tpFPS + tpDXS.


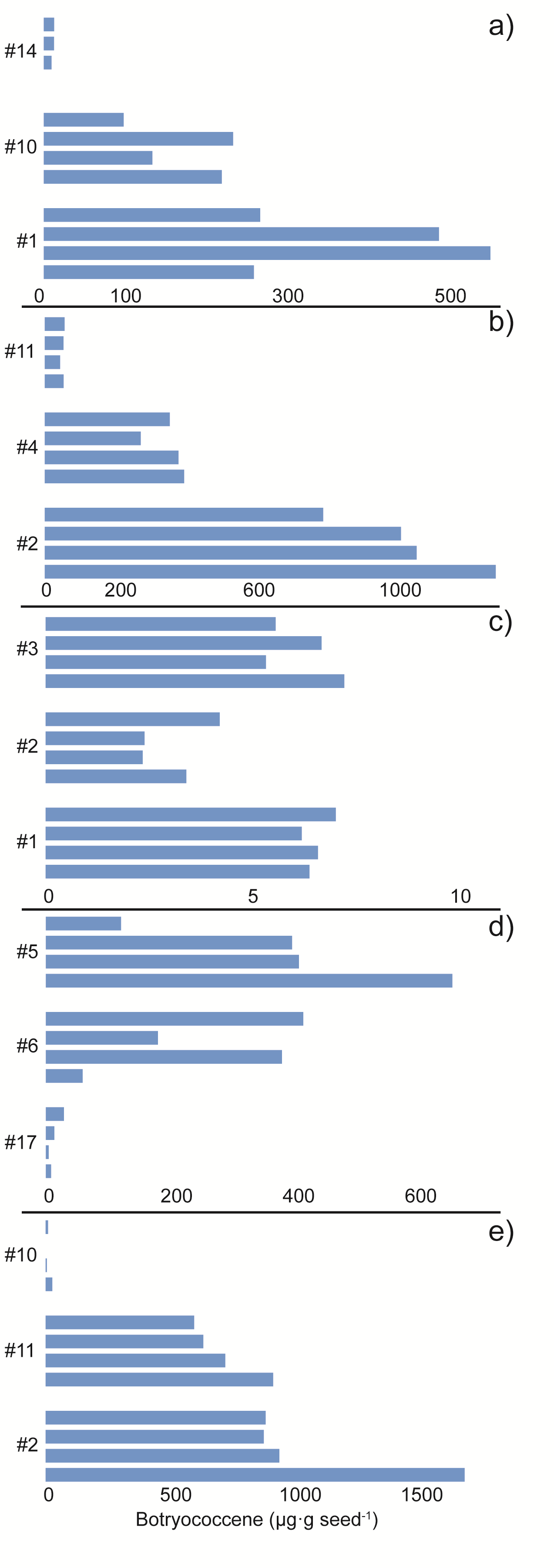


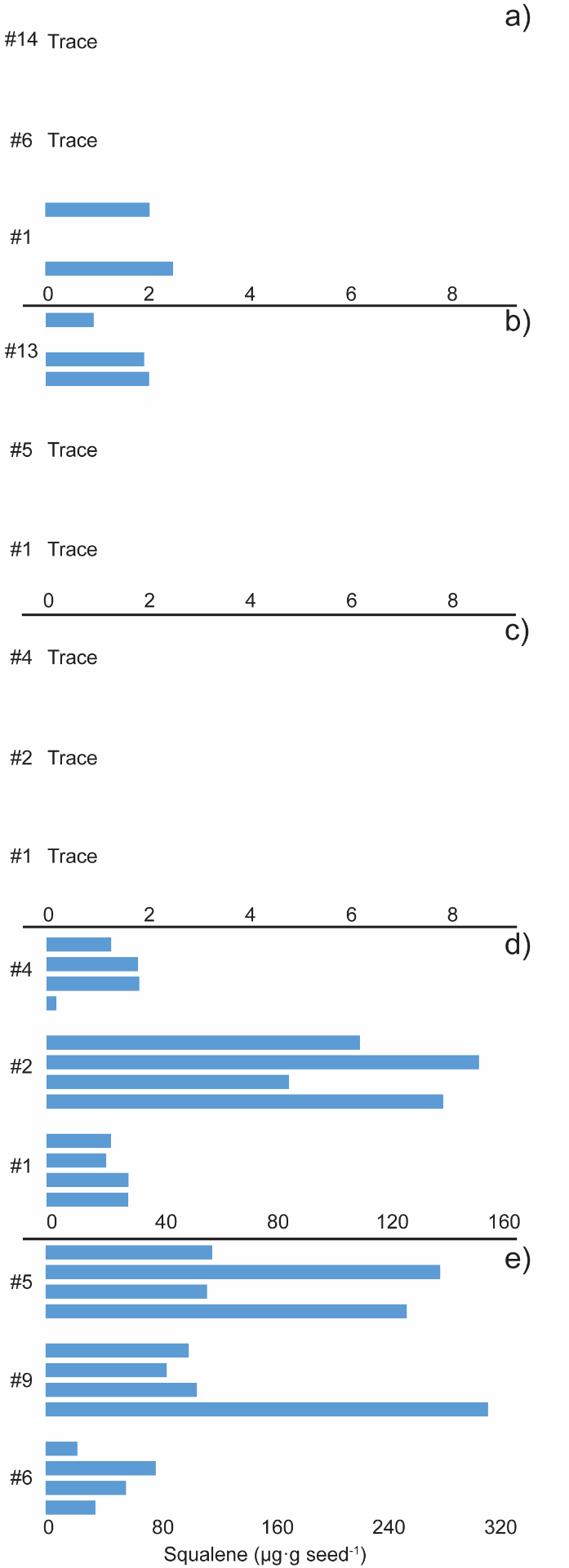
Figure S2. Squalene content (μg·g seed^-1^) of four independent T_3_ seed lots. These T_3_ seed lots were derived from four sister T_2_ plants from an independent transformation event of the designated construct (see Figure 2 for schematic for how tissue was generated). Bars represent a single determination from 10-25 mg of seeds. Trace indicates amounts too small to integrate given the sample size. Three independently generated lines are shown for each construct, representing low, medium, and high accumulators. Engineered enzymes are: a) SQS, b) SQS + FPS, c) tpSQS, d) tpSQS + tpFPS, e) tpSQS + tpFPS + tpDXS.

Figure S3. Example of phenotypes complicating segregation analsys. A normal line, which exhibited uniform resistant engineered plants (tpBS + tpFPS 13-3, top), segregating in a single gene ratio (3:1), versus a line exhibiting multiple phenotypes (tpBS + tpFPS 17-6, bottom), complicating segregation analysis.


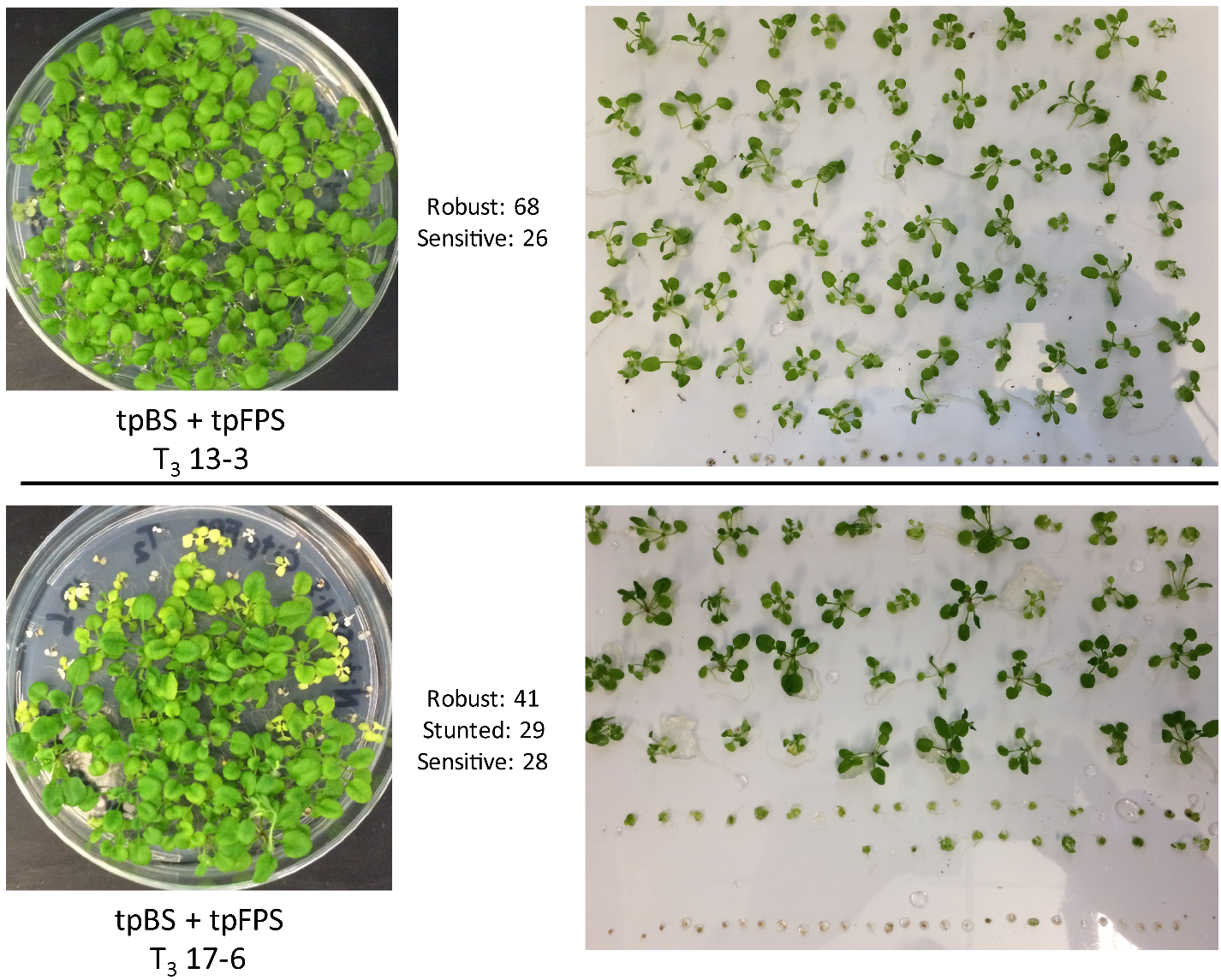


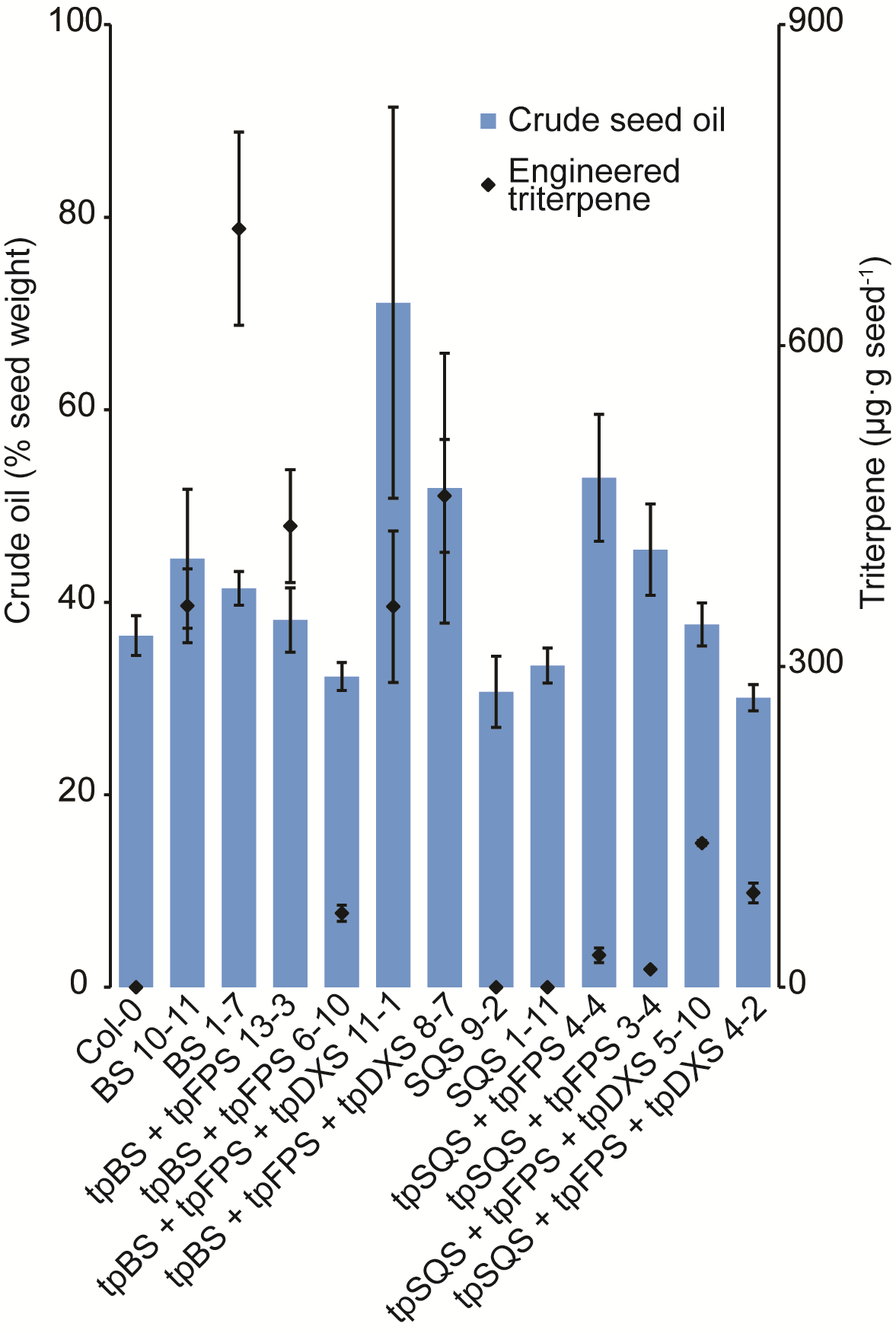


Figure S4. Crude seed oil (% seed weight) and triterpene values from indicated T_3_ seed lots. Crude seed oil (indicated as % seed weight) is indicated with blue bars (±SE; *n*=3, no significance of transgenic lines versus Col-0 at *p-*value < 0.05, Student’s *t*-test). See the Experimental Procedures for how crude seed oil was determined. Triterpene accumulation (botryococcene for BS constructs and squalene for SQS constructs) is indicated with black diamonds (±SE, *n*=3).
